# Quantifying the Relationship between Land Use Intensity and Ecosystem Services’ Value in the Hanjiang River Basin: A Case Study of the Hubei Section

**DOI:** 10.3390/ijerph191710950

**Published:** 2022-09-02

**Authors:** Hui Yang, Liang Zheng, Ying Wang, Jiangfeng Li, Bowen Zhang, Yuzhe Bi

**Affiliations:** 1 School of Public Administration, China University of Geosciences, Wuhan 430074, China; 2 Changjiang Institute of Survey, Planning, Design and Research, Wuhan 430014, China; 3 Key Laboratory of Changjiang Regulation and Protection of Ministry of Water Resources, Wuhan 430014, China

**Keywords:** ecosystem services’ value (ESV), land use intensity, spatiotemporal characteristics, spatial correlations, driving factors, Hanjiang River Basin (HRB)

## Abstract

An increased land use intensity due to rapid urbanization and socio-economic development would alter the structure and function of regional ecosystems and cause prominent environmental problems. Revealing the impact of land use intensity on ecosystem services (ES) would provide guidance for more informed decision making to promote the sustainable development of human and natural systems. In this study, we selected the Hanjiang River Basin (HRB) in Hubei Province (China) as our study area, explored the correlation between land use intensity and ecosystem Services’ Value (ESV), and investigated impacts of natural and socio-economic factors on ESV variations based on the Geographical Detector Model (GDM) and Geographically Weighted Regression (GWR). The results show that (1) from 2000 to 2020, land use intensity in HRB generally showed an upward trend, with a high spatial agglomeration in the southeast and low in the northwest; (2) the total ESV increased from 295.56 billion CNY in 2000 to 296.93 billion CNY in 2010, and then decreased to 295.63 CNY in 2020, exhibiting an inverted U-shaped trend, with regulation services contributing the most to ESV; (3) land use intensity and ESV had a strong negative spatial correlation, with LH (low land use intensity vs. high ESV) aggregations mainly distributed in the northwest, whereas HL (high land use intensity vs. low ESV) aggregations were located in the southeast; (4) natural factors, including annual mean temperature, the percentage of forest land, and slope were positively associated with ESV, while socio-economic factors, including GDP and population density, were negatively associated with ESV. To achieve the coordinated development of the socio-economy and the environment, ES should be incorporated into spatial planning and socio-economic development policies.

## 1. Introduction

Land provides space for human activities and supports terrestrial ecosystem services (ES) that are essential for human survival and development. ES are the goods (e.g., food, water, etc.) and services (e.g., air purification, waste treatment, etc.) that ecosystems provide to human society, which can be broadly classified into four categories, i.e., supply services, regulation services, support services, and cultural services [1,2]. During the process of rapid urbanization and industrialization, humans have drastically transformed the landscape from natural surfaces (such as forest land and water areas) to surfaces employed for artificial uses (such as cultivated land and built-up areas), and the land use intensity has substantially increased, which greatly weakens the provision of vital ES by ecosystems [3,4]. In light of this, promoting the coordination between humans and ecosystems has become a hot topic for both governments and academia. For example, the United Nations identified Goal 7: Ensure environmental sustainability as an indicator of the Millennium Development Goals and suggested that it be incorporated into country policies and programs to reverse the loss of ES. Faced with prominent environmental issues, the 18th National Congress of the Communist Party of China (CPC) in 2012 proposed the ecological civilization construction strategy, which emphasized harmony between human and nature [5].

Research on ES has begun to flourish since Costanza et al. (1997) published the famous paper, “The Value of the World’s Ecosystem Services and Natural Capital”, which classified the global ES into 17 types and estimated their economic values [1]. Xie et al. (2003 and 2008) [6,7] built upon Costanza et al. (1997) and proposed an evaluation method suitable for assessing the economic value of terrestrial ES in China based on surveys of over 200 Chinese ecologists [8]. Much of the literature on ES has focused on the evaluation of ES value (ESV) [9,10,11], the driving mechanism of ES variation [12,13,14], the integration of ES in landscape planning and decision making [15,16], and analysis of ES synergies and tradeoffs [17,18]. Recent research has begun to investigate the coupling coordinative relationship between ES and socio-economic development, such as sustainable development [19,20], human activities’ intensity [21], urbanization [22], etc.

From the perspective of land use, Xi, et al. [23] analyzed the spatiotemporal characteristics of the ESV of island cities based on land use/cover and predicted future ESV. Rahman and Szabó [24] analyzed the impact of land use/cover change (LUCC) on the value of urban ES in Dhaka, Bangladesh, and found that water areas contributed the most to ESV. However, less attention has been drawn to the relationship between land use intensity and ESV. Land use intensity reflects the extent to which land has been developed and utilized by human activities. Some studies take it as an indicator of land use efficiency [25], while others use it to measure the development of regional land parcels [26]. This study uses it to measure the degree to which different land uses are developed by human beings. The existing research on land use intensity has been widely studied in the literature, including the intensity of cultivated land use [27], the response of land use intensity to urbanization [26], and the relationship between land use intensity, the ecological environment [28], and biodiversity [29]. In this study, we aim to explore spatial correlations between land use intensity and ESV. 

According to previous studies on the driving mechanism of ES change, the evolution of regional ES is affected by a combination of natural and human factors [30]. Natural factors include precipitation, temperature, and vegetation coverage [31]. The anthropogenic aspect comprises the effects of human-induced climate change and LUCC, as well as the effects of economic development and human activities [32,33]. The selected anthropogenic factors primarily consist of population, urbanization rate, GDP, etc. The impacts of these factors vary widely due to regional differences [34,35]. Understanding the influencing factors and driving mechanisms of regional ES in different locations is essential for targeted plans and measures to achieve environmental protection and sustainable development [36]. 

As the largest tributary of the Yangtze River, the socio-economic position of the Hanjiang River Basin (HRB) is crucial for the Yangtze River Basin. With the development of the Yangtze River Economic Belt, especially the opening of the middle route of the South-to-North Water Diversion Project, the ecosystem of the Hanjiang River is under great threat [37]. The reduction in the water volume and the destruction of vegetation in the upper reaches of the Hanjiang River directly affect the water quality and hydrological conditions in the middle and lower reaches, i.e., the HRB in Hubei Province. The Danjiangkou Reservoir in Hubei Province is the core water source of the middle route of the South-to-North Water Diversion Project [38], and the water transfer has a great impact on the production and ecology of the middle and lower reaches of the Hanjiang River. Furthermore, the HRB in Hubei Province plays a very important role in the development of the province, with more than 50% of its population and GDP being distributed in the HRB. Hence, decision makers attach great importance to the development and implementation of policy in Hubei Section of HRB. Over the past two decades, rapid urbanization and over-reclamation of cultivated land have resulted in an imbalance of land use structure in the HRB of Hubei Province. This imbalance is primarily manifested by the continuous expansion of built-up land at the expense of high-quality cultivated land, forest land, and water area, resulting in resource depletion and environmental pollution [39]. Due to increased human activities, the ability of ecosystems in HRB to self-regulate has degraded.

Some scholars have investigated the ES of the HRB. For example, Li, et al. [40] took the upper Hanjiang River as their study area and examined the changes of water-related ES, such as soil conservation and flood control services, as a result of climate change. Qi et al. [41] explored the role of forest restoration in ES in the HRB and found a positive impact. Yu, et al. [42] explored the evolution of the social-ecological system in the Hubei Section of the HRB and found that resources and the economy were important driving forces of the change in social-ecological systems, and that human activity played a leading role in its evolution. Existing studies in the HRB have focused on a single type of ES from a micro perspective, and the majority of the study areas are located in the upper reaches. Few studies have examined the overall ES in the basin and the correlation between land use intensity and ES, as well as the driving force of ES, particularly in the middle and lower HRB reaches. Additionally, as one of the most representative human activities, the South-to-North Water Diversion Project has put great pressure on the environment and society in the middle and lower reaches of the Hanjiang River. Our study period ranged from 2000 to 2020, which allowed us to examine changes in the regional environmental conditions before and after the implementation of the South-to-North Water Diversion Project in 2014. It is of great value to investigate the relationship between land use intensity and ES in this region for the sustainable development of human and the environment. Thus, in this study, we selected the HRB in Hubei Province as our study area to investigate the responses of ESV to changes in land use intensity. This study has four specific research objectives: (1) to identify the spatiotemporal changes of land use intensity, (2) to assess the spatiotemporal evolution of ESV, (3) to analyze the spatial correlations between land use intensity and ESV, and (4) to reveal the driving factors affecting ESV changes in the Hubei section of the HRB from 2000 to 2020.

## 2. Materials and Methods

### 2.1. Study Area

**Figure 1:**
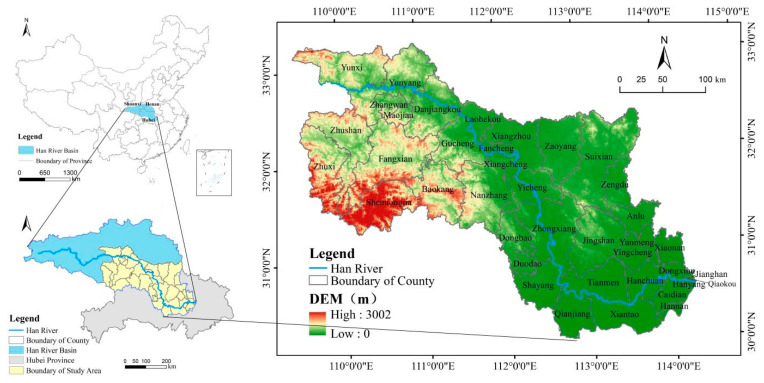
Location of the study area.

The Hanjiang River originates in the Qinling Mountains; flows primarily through Shaanxi, Henan, and Hubei provinces, and has a total length of 1567 km and a total area of 15.9 × 10^4^ km^2^. It joins the Yangtze River from west to east and is the largest tributary of the Yangtze River. The landform of the HRB descends a total of 1964 m from mountains to plains [43]. Located in the subtropical monsoon climate zone, the HRB has an annual average precipitation of 700–1800 mm, an annual average temperature of 14 °C, and a relatively high vegetation coverage rate [44]. After passing through Baihe County, the Hanjiang River enters Hubei Province from Yunxi County, turns southeast at Danjiangkou, and passes through Xiangyang, Yicheng, Zhongxiang, and other counties on its way to Wuhan City, where it joins the Yangtze River. The HRB in Hubei Province, encompassing nearly the middle and lower reaches of the Hanjiang River, was selected as our study area (Figure 1).

### 2.2. Data Sources

The land use raster dataset with a 100 m resolution for the years 2000, 2010, and 2020 was downloaded from the Data Center for Resources and Environmental Sciences, at the Chinese Academy of Sciences (RESDC) (http://www.resdc.cn, accessed on 5 May 2022). Annual mean temperature, annual mean precipitation, slope, GDP, and population density were also obtained from RESDC. The distances to the county center, water system, and road system were calculated using the Euclidean distance tool in ArcGIS 10.3 software (ESRI, Environmental Systems Research Institute, Redlands, CA, USA). ArcGIS 10.3 was also used to calculate the area of different land use types in each county. All datasets were converted into the same coordinate system and the same pixel size (100 m × 100 m).

### 2.3. Methods

#### 2.3.1. Calculation of Land Use Intensity

Land is the material basis for the survival and development of human society. Land use intensity reflects the extent to which land resources are developed and utilized by human beings [45]. Referring to the method of land use intensity proposed by Zhuang et al. [46] and the graded assignments of land use type (Table 1), the land use intensity can be calculated with the following formula:

(1) $$L=100\times \overset{n}{\underset{i=1}{\sum }}R_{i}\times A_{i}/A_{t}$$

where L is the land use intensity, n is the number of land use types, $R_{i}$ is the grade factor of the i-th land use type, $A_{i}$ is the area of the i-th land use type, and $A_{t}$ is the total area of all land use types.

#### 2.3.2. Assessment of Ecosystem Services’ Value

The evaluation method proposed by Costanza et al. [1] and adapted by Xie et al. [6,7] for China’s ecosystems has been widely adopted due to its high operability and convenient method of data acquisition [8]. In general, ES is classified into four categories, i.e., supply services, regulation services, support services, and cultural services, which can be further divided into nine subtypes (Table 2). Based on the equivalent value per-unit area of ES proposed by Xie et al. in 2008, we adjusted the economic value of a standard equivalent factor and calculated the ESV of the study area. According to the functions and characteristics of land use types, we matched forest land with forest in Xie et al.’s classification system, cultivated land with farmland, water area with rivers/lakes, unused land with desert, and assigned built-up land an ESV of zero [47]. It should be noted that the economic value of a standard equivalent factor equals 1/7 of the average market value of grain production. Considering the grain yield per-unit area of the study area and the average grain prices in 2000, 2010, and 2020, the equivalent factor value was calculated as 1881.45 CNY/hm^2^. The formula for estimating the total ESV in the study area is as follows:

(2) $$\mathrm{ESV}=\sum^{\;}k\cdot E_{i}\times A_{i}$$

where k is the equivalent factor value of ES; $E_{i}$ is the ESV per-unit area of the i-th land use type; $A_{i}$ is the area of the i-th land use type.

#### 2.3.3. Hot Spot Analysis

Getis–Ord Gi* is an index of local spatial autocorrelation used to explore the spatial clustering of high values (hot spots) or low values (cold spots) of spatial variables [48]. The output can be represented with Z-score, p-value, and confidence level. We used the Getis–Ord Gi* tool in ArcGIS 10.3 software to analyze the hot spots and cold spots of ESV in the study area. See Appendix A.1.1 for more detailed description of the hot spot analysis method.

#### 2.3.4. Bivariate Spatial Autocorrelation Model

Spatial autocorrelation refers to the statistical correlation of a certain attribute value of geographic objects with spatial location differences. Generally, the closer the two values are, the greater the correlation. Spatial autocorrelation analysis is an important indicator to measure the aggregation or discrete distribution of spatial elements, and is generally described by global Moran’s *I* and local Moran’s *I* [49]. The global autocorrelation tests the spatial vergence pattern of the spatial variables over the entire research range, while the local spatial autocorrelation captures the correlation of the variables in different regional units [50]. In this study, the bivariate spatial autocorrelation model was used to investigate the spatial correlation between land use intensity and ESV using GeoDa 1.18 software. Moran scatter plots and LISA cluster maps were adopted to analyze local spatial correlation and reflect the significance level of spatial correlation. See Appendix A for a more detailed description of the spatial autocorrelation model (Appendix A.1.2).

#### 2.3.5. Analysis of the Driving Mechanism

It is well-established in the literature that changes in ES are driven by both natural and human factors. The natural dimension includes climate factors (e.g., temperature and precipitation), topography (e.g., slope), and vegetation (e.g., the proportion of forest land), which are found to directly affect ES supply and demand [51]. Human activities can be represented by socio-economic factors, including GDP, population density, and percentage of built-up land [35], which are often used to measure regional economic development and urbanization level. In general, the higher the GDP, population density, and percentage of built-up land, the higher the degree of human interference with the ecosystem. In addition, geographic locations, such as distance to the county center, road, and water system, also have impacts on ES, mainly affecting the spatial patterns of ESV [52]. 

Based on the above analyses, ten driving factors were selected as potential drivers of ESV change (Table 3). Then, Geographical detector model (GDM) and Geographically Weighted Regression (GWR) were used to detect and analyze the driving forces that affect the ESV. GDM can detect not only the influence of driving factors but also their interactions. The GWR model can be used to explore the directions and spatial distributions of the impacts of each driving factor.

##### Geographical Detector Model

GDM is comprised of risk detection, factor detection, ecological detection, and interactive detection, which can be used to detect spatial variation and identify potential influencing factors [53]. The GDM has been widely used in many fields, including social-economy and the ecological environment [51,54]. See Appendix A.1.3 for a more detailed description of the GDM. 

##### Geographically Weighted Regression

GWR is an extension of the traditional regression analysis method that can estimate data with spatial autocorrelation and reflect the spatial heterogeneity of parameters [55]. The GWR can reveal the direction and magnitude of influence of each factor in different locations [56]. See Appendix A.1.3 for more detailed description of the GWR model.

**Figure 2:**
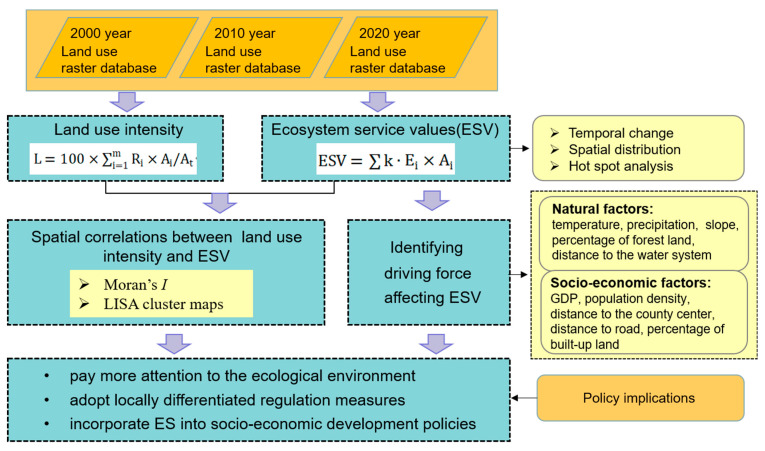
The framework of this study.

The flow chart of the study is illustrated in Figure 2.

## 3. Results

### 3.1. Spatiotemporal Characteristics of Land Use Intensity

**Figure 3:**
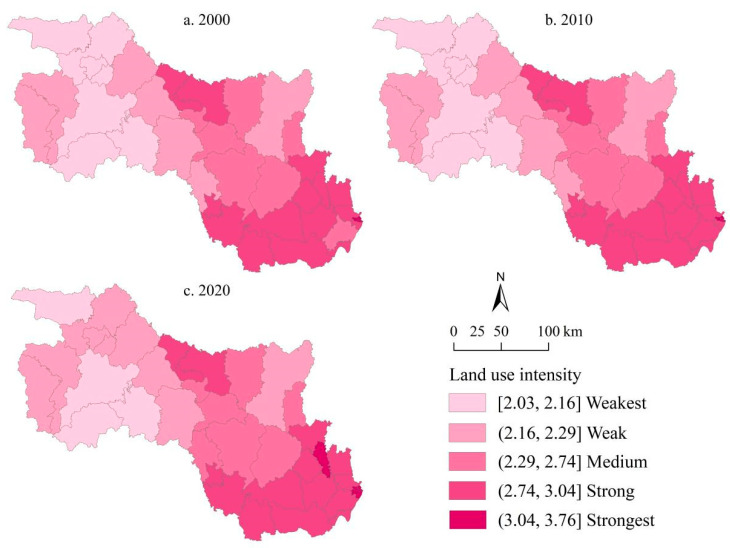
The spatial pattern of land use intensity in the HRB of Hubei Province for (**a**) 2000; (**b**) 2010; (**c**) 2020.

Figure 3 depicts the land use intensity for each county in the HRB in Hubei Province from 2000 to 2020. High-value areas of land use intensity were primarily concentrated in the southeast, where economic development was relatively advanced, whereas low-value areas were primarily distributed in the northwest, where the ecological environment was superior and development was relatively lagging. This result indicates that land use intensity has a spatial pattern of “centralized distribution”. A high-value central area was formed by Jianghan, Hanyang, and Qiaokou districts of Wuhan City. Other counties close to the high-value area also had higher levels of land use intensity. The land use intensity decreased gradually from the county center to the county periphery as the distance increased.

Overall, land use intensity showed a slight upward trend from 2000 to 2020. The counties with the most notable increases were located in the southeast of the study area. For example, the land use intensity of Caidian District changed from weak to medium, and Hanyang District and Qiaokou District changed from strong to strongest. In addition, the disparity in land use intensity between counties was narrowing, and land use intensity in the whole study area remained relatively stable.

### 3.2. Spatiotemporal Characteristics of ESV

#### 3.2.1. Temporal Change of ESV

Forest land and cultivated land in the study area constituted the largest share of the landscape, accounting for 48.88% and 38.71% of the total area in 2020, respectively, followed by water area and grassland (Table 4). From 2000 to 2020, the area of cultivated land and forest land decreased the most, by 1119.04 km^2^ and 245.83 km^2^, respectively. The total ESV of the HRB in Hubei Province was 2955.62 × 10^8^ CNY, 2969.34 × 10^8^ CNY, and 2956.30 × 10^8^ CNY in 2000, 2010, and 2020, respectively (1 CNY = 0.1450 US dollar in 2020), with an inverted U-shaped trend of first increasing and then decreasing. Overall, the total ESV increased by 68 million CNY, representing a change rate of 0.02%. Table 4 shows that the ESV of forest land accounted for the largest proportion, greater than 70% throughout the study period, followed by cultivated land and water area, with the highest proportions in 2000 and 2020, respectively, being 16.24% and 12.14%.

**Figure 4:**
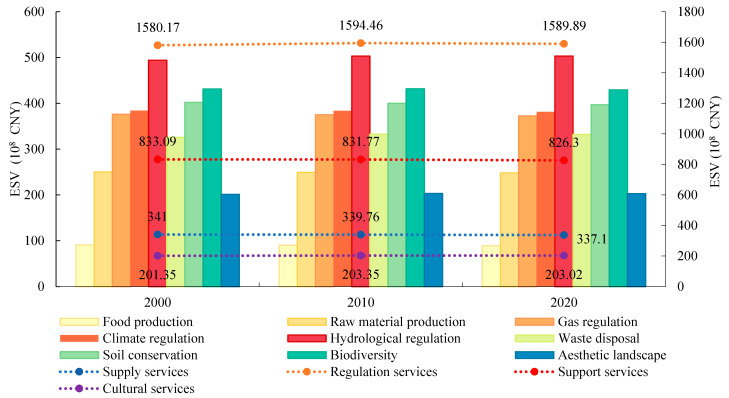
ESV of different ecosystem service types from 2000 to 2020.

Figure 4 exhibits the changes in the ESV of different categories of ES in the study area from 2000 to 2020. These changes were minor, and the structure of the ESV remained relatively stable. The regulation services provided the largest value, reaching up to 1589.89 × 10^8^ CNY in 2020. The ESV of cultural services was the lowest, at only 203.02 × 10^8^ CNY in the same year. Among the nine subtypes of ES, the value of hydrological regulation services was the largest, at 503.32 × 10^8^ CNY in 2020, followed by biodiversity, soil conservation, and climate regulation services, with values of 429.25 × 10^8^ CNY, 397.04 × 10^8^ CNY, and 381.49 × 10^8^ CNY, respectively. During the study period, the hydrological regulation and waste disposal services increased by 9.23 × 10^8^ CNY and 6.35 × 10^8^ CNY, respectively, whereas all other types of ES showed a slight decline.

#### 3.2.2. Spatial Distribution Characteristics of ESV

**Figure 5:**
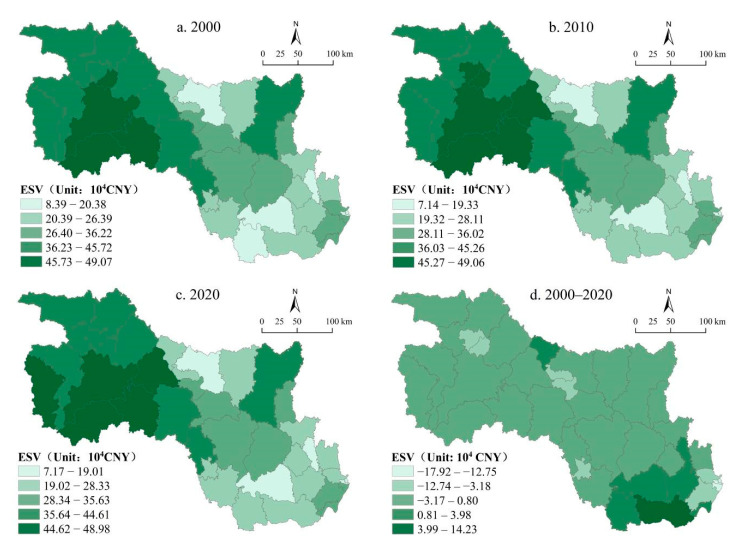
Spatial distribution of ESV (**a**–**c**) and the change rates (**d**) in the HRB of Hubei Province for 2000, 2010, and 2020.

We used the ArcGIS 10.3 software to spatially visualize ESV and then classified it into five grades using the natural breaks method. As shown in Figure 5, the ESV exhibited clear spatial differentiation. From 2000 to 2020, the high-value areas of ESV were mainly distributed in the west and northwest of the study area, especially in Maojian, Fangxian, Baokang, and Shennongjia. The higher value of ESV was the result of the presence of water bodies, forests, and vegetation in these counties. The low-value areas were mainly distributed in the southeastern areas, where cultivated land and the economically developed areas were concentrated. Overall, the spatial distribution of ESV was high in the northwest and low in the southeast. Figure 5d depicts the spatial distribution of ESV change rates from 2000 to 2020, indicating that the ESV decreases in the majority of counties within the study area, with change rates ranging from −3.18% to 0.80%. The Qiaokou, Jianghan, and Hanyang districts experienced the largest declines in ESV, with respective change rates of −17.92%, −14.53%, and −12.75%; Xiantao witnessed the largest growth in ESV, which was up to 14.23%. The spatial distribution of the ESV change rates was closely related to the land use structures and regulation policies of different counties.

**Figure 6:**
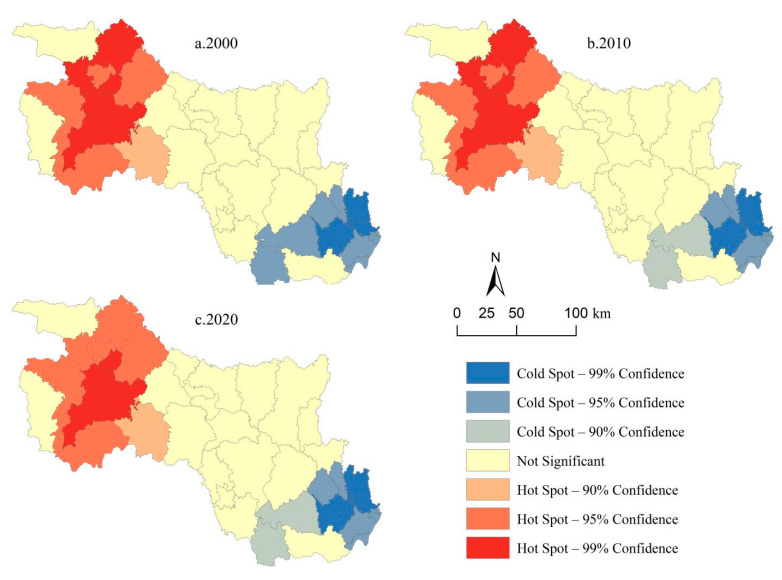
Spatial agglomeration characteristics of ESV in the HRB of Hubei Province for (**a**) 2000, (**b**) 2010, and (**c**) 2020.

Based on a hot spot analysis, we further revealed the spatial agglomeration characteristics and evolution of ESV in the HRB of Hubei from 2000 to 2020 (Figure 6). The spatial agglomeration of ESV was insignificant in nearly two-thirds of the study area, and the significant regions were mainly distributed in the northwest and southeast. The high-value (hot spot) agglomeration areas of ESV were mainly distributed in the northwest, whereas the low-value (cold spot) agglomeration areas were mainly distributed in the southeast, forming the spatial pattern of high in the northwest and low in the southeast. From 2000 to 2020, the range of hot spot and cold spot agglomerations remained stable, with the confidence level of hot spots for several counties reducing from 99% to 95%, while the strength of the significance weakened.

### 3.3. Spatial Correlations between Land Use Intensity and ESV

**Figure 7:**
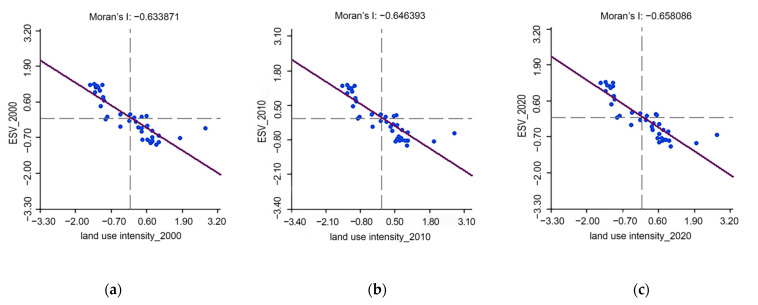
Moran scatter plots of land use intensity with ESV in the HRB of Hubei Province for (**a**) 2000, (**b**) 2010, and (**c**) 2020.

**Figure 8:**
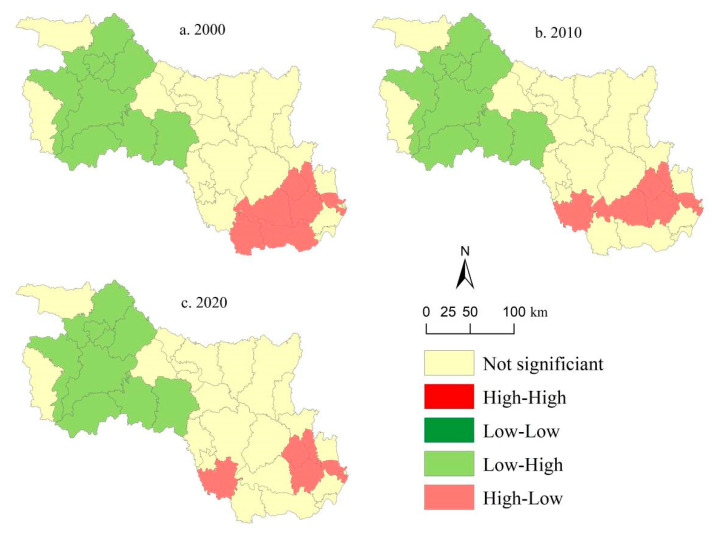
LISA cluster maps between land use intensity and ESV in the HRB of Hubei Province for (**a**) 2000, (**b**) 2010, and (**c**) 2020.

The results from the global bivariate Moran’s *I* revealed significant negative spatial correlations between land use intensity and ESV, regardless of the ES type (all Moran’s *I* values < 0) (Figure 7). The global bivariate Moran’s *I* in 2000, 2010, and 2020 was −0.63, −0.65, and −0.66 respectively; the majority of the values are in the second and fourth quadrants. The absolute values of Moran’s *I* from 2020 to 2020 also indicated that the negative correlation was becoming increasingly stronger. This strongly demonstrates that the deepening of land use intensity will lead to the decrease in ESV in the HRB. Figure 8 presents the bivariate local spatial autocorrelation LISA aggregation maps between land use intensity and ESV at the county level for the years 2000, 2010, and 2020. The clustering pattern of the correlation between land use intensity and ESV was obvious, and there were only two types of spatial correlations between the two, namely, LH (low land use intensity vs. high ESV) and HL (high land use intensity vs. low ESV). The LH areas were mainly concentrated in the northwest of the study area, and the HL areas were in the southeast. During the study period, the spatial correction between land use intensity and ESV exhibited a slight shift in its clustering pattern. From 2000 to 2010, both Qianjiang and Xiantao cities changed from HL to insignificant, and the changes in Shayang County and Qiaokou District exhibited the opposite change pattern (Figure 8a,b). Tianmen City changed from HL to not-significant land use during 2010–2020 (Figure 8c).

### 3.4. Spatial Variability of Driving Factors on ESV Changes

#### 3.4.1. Results of GDM

The factor detection module of the GDM was used to quantify the impacts of natural and socio-economic factors on ESV (Table 5). Among the natural factors, the percentages of forest land (X4) and slope (X3) had the greatest explanatory power (with q values of 0.87 and 0.81, respectively) for ESV spatial variation. Regarding the socio-economic factors, the explanatory power of the percentage of built-up land (X10) and GDP (X6) on ESV variations was 0.74 and 0.65, respectively, and both were significant at the 1% level. Only the precipitation (X2) and distance to the water system (X5) did not have significant effects on ESV.

According to the results of the interaction detector (Table 6), there was no mutual weakening in the 45 pairs of interaction combinations, indicating that the impact of multiple driving factors on ESV is greater than that of a single factor. Except for the interaction results of the precipitation (X2) and the distance to a water system (X5), which are of the nonlinear enhancement type, the interaction results of the other bivariate combinations were enhanced. For example, the interaction between the percentage of forest land (X4) and the distance to a road (X9) explained the ESV changes with the greatest explanatory power (q value = 0.94), followed by the interaction between the percentage of forest land (X4) and the distance to a water system (X5), as well as the percentage of forest land (X4) and GDP (X6), with a q value of 0.92. The results of the interactive detection further verify that the percentage of forest land played a leading role in the spatial distribution of regional ESV changes.

#### 3.4.2. Results of GWR

Table 7 depicts the performance parameters of the GWR and the ordinary least squares regression (OLS) model, which suggest that the GWR model has a better predictive ability than the OLS, as it had higher R^2^ and adjusted R^2^ values, and a lower AICc value.

**Figure 9:**
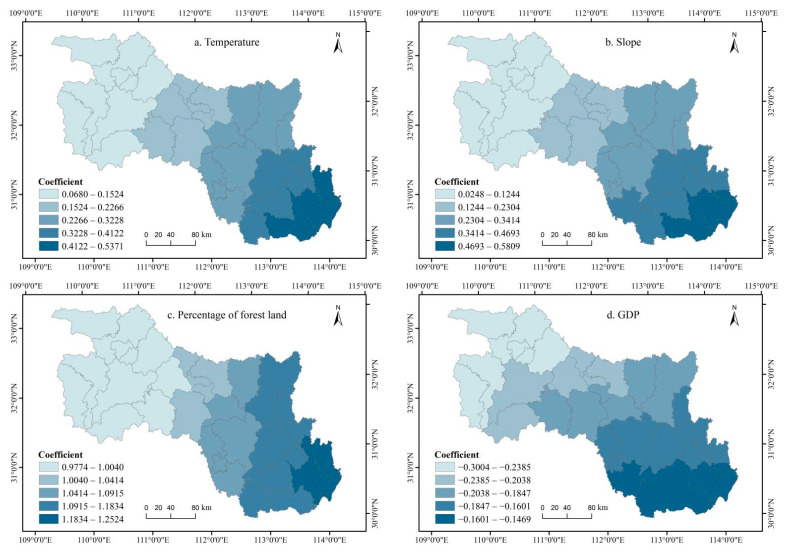

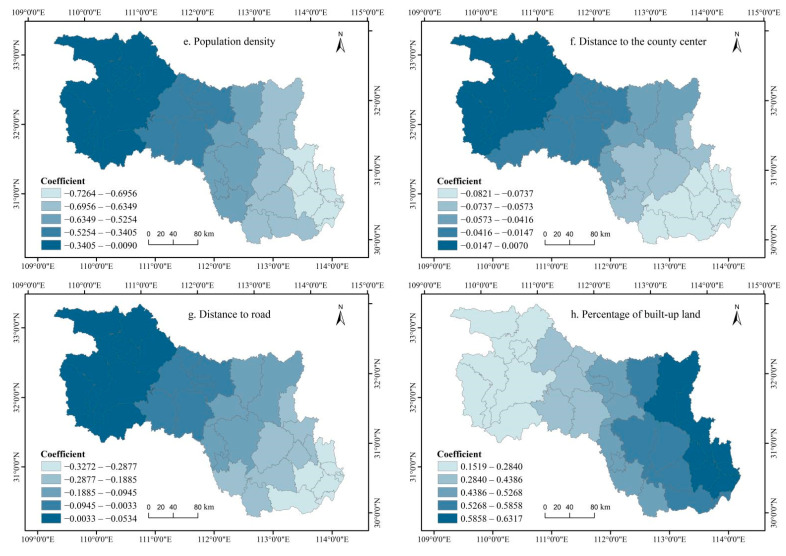
Spatial distribution of regression coefficients in GWR.

Since the GDM found that precipitation (X2) and the distance to a water system (X5) have no significant impact on ESV, we removed these two factors and only explored the spatial distribution of regression coefficients for the remaining eight factors. As illustrated in Figure 9, each driving factor had an obvious spatial heterogeneity, indicating that the same factor had different impacts on the ESV at different spatial locations, and there was a significant spatial non-stationarity. Among the natural factors, ESV had a significant positive correlation with temperature, slope, and the percentage of forest land, with a higher correlation coefficient in the southeast and a lower correlation coefficient in the northwest (Figure 9a–c). This indicates that the enhancement of these factors contributes to the improvement of ESV. In terms of socio-economic factors, the regression coefficients of GDP and population density were both negative, demonstrating that an increase in these factors will weaken the ESV. The distance to the county center and the distance to a road had negative correlations with ESV in most regions, and only a few counties in the southeast had a positive correlation. The absolute values of the influence of GDP and percentage of built-up land were consistent, with high values in the southeast and low values in the northwest, which were spatially similar to the driving forces of natural factors. The effects of population density, distance to county center, and distance to a road on ESV were not only consistent in their correlation but were also similar in distribution, showing values of high in the northwest and low in the southeast (Figure 9d–h). In conclusion, the order for the size of the impacts of the eight driving factors on ESV was as follows: percentage of forest land > population density > percentage of built-up land > slope > temperature > GDP > distance to a road > distance to the county center (Figure 9).

## 4. Discussion

### 4.1. Spatial Relationship between Land Use Intensity and ESV

From 2000 to 2010, ESV experienced an inverted U-shaped trend. The changes in ESV were mainly due to unreasonable land use planning and low land utilization rate, which led to the rapid growth of built-up land at the expense of forest land, cultivated land, and grassland. Land use intensity has a significant negative relationship with ESV [57]. Socio-economic development led to dramatic changes in land use structure, and the increase in land use intensity was the direct cause of ESV degradation [58,59]. To further explore the characteristics of the spatial correlation between land use intensity and ESV, we used the bivariate spatial autocorrelation method to study the spatial relationship between the two. During the study period, the Moran’s *I* was entirely negative, and its absolute value showed a trend of increasing (Figure 7). This indicated that the negative correlation between land use intensity and ESV in the study area had become more pronounced over time, which was in line with prior studies on the relationship between LUCC and ESV [60,61]. The intensification of land use was mainly manifested in the increasing expansion of built-up land; the continuous occupation of cultivated land, forest land, and grassland; the extensive land utilization; and the low land utilization. Consequently, the ES provided by ecosystems was deteriorating. The LISA cluster maps revealed a significant spatial correlation between land use intensity and ESV (Figure 8). The LH areas were mainly distributed in the hilly and mountainous areas with higher terrain and steeper slopes in the northwest of the study area (such as the Shennongjia Mountain, Wudang Mountain, etc.), while HL areas were mainly distributed in the middle and lower reaches of the Yangtze River with flat terrain and dense lakes in the southeastern part of the study area, particularly in Wuhan—which is known as the “city of a thousand lakes”—and the surrounding cities. This was due to the mountainous and hilly terrain in the northwest region, which made land development difficult and costly. In addition, the area of forest land in this region is large, which provides human society with crucial ES such as biodiversity maintenance, climate regulation, and ecological conservation; thus, the ESV was high. The situation in the southeast was the opposite of that in the northwest. The unique natural environment created the conditions for high-density population agglomeration and high-intensity land development, resulting in the disorderly spread of built-up land and the occupation of ecological lands, especially water bodies and cultivated land; thus, the ESV in this region was at a low level.

For the ecosystem in the HRB of Hubei Province, the middle route of the South-to-North Water Diversion Project is undoubtedly one of the most representative human activities. The opening of the project had a great impact on the aquatic ecological environment, climate conditions, and people’s production and life in this area. Due to the reduction in the water volume in the basin, there are problems such as the decline in the water purification capacity, the reduction of aquatic organisms, the decrease in aquatic environmental carrying capacity, and the deterioration of the aquatic ecological environment. At the same time, the industrial and agricultural sectors—with a great demand for water—are facing a water shortage, and the industrial structure is changing, which will affect the production and lifestyles of people in the region [62]. The construction of the project also brought about the problem of immigration. The change from farmland to settlement and the establishment of new residential areas for immigrants are among the reasons for the expansion of built-up land and the reduction of cultivated land and forest land [63].

### 4.2. Identifying Driving Factors Affecting ESV

According to the Geographical Detector Model (GDM) (Table 5 and Table 6), the percentage of forest land had the largest positive effect on ESV, which was consistent with previous studies where regions with a large forest area provided greater regulation and support services and had higher ESV [64,65]. Thus, strengthening the protection of forest land and increasing the forest coverage rate of each county is of great significance for promoting regional climatic improvement and alleviating the greenhouse and heat island effect. The results of the Geographically Weighted Regression (GWR) analysis further revealed the dominant role of natural factors with respect to ESV and the growing trend of socio-economic factors (Figure 9). Natural factors had positive impacts on ESV and the regions with more favorable natural conditions had larger ESVs. However, the areas with high driving coefficients of natural factors were concentrated in the economically developed counties in the southeast. This was because better economic conditions in many regions come at the expense of environmental degradation. Therefore, if the protection of the natural environment of counties in this region is enhanced, based on the same input conditions, the increase in ESV must be much higher than those of the regions with relatively poor socio-economic conditions but a superior ecological environment. This can also explain the “high in the northwest and low in the southeast” distribution of socio-economic factors such as GDP. Nevertheless, not all socio-economic factors had the same distribution of driving forces as GDP. For example, the distribution of the driving coefficients of population density, the distance to the county center, and the distance to a road was “low in the northwest and high in the southeast”. This indicated that the improvement of socio-economic conditions had less of an effect on the ESV of the undeveloped counties in the northwest. For these regions, the improvement in socio-economic conditions would not result in a substantial decrease in ESV. However, for the more economically developed and densely populated southeastern regions, the lack of environmental protection would increase regional environmental pressure and lead to a rapid decline in ESV [66]. The influence of the distance to the county center and the distance to a road on ESV was predominantly negative, except for a few northwest counties. 

In conclusion, ESV was influenced by both natural and socio-economic factors in an interactive way [67]. Although multiple types of ES are provided by natural systems to maintain human welfare, human activities have altered the structure and function of ecosystems, which further affect the provision of vital ES by ecosystems [68]. Therefore, it is important to protect and restore the crucial ecosystems through landscape planning, regulative policies, and environmental programs. 

### 4.3. Policy Implications

The increased land use intensity during rapid urbanization and social-economic development has inevitably degraded the ecological environment [69,70], as evidenced by the reduction of forest land and cultivated land, air pollution, severe climate change, waste of land resources, etc. The existing land use planning and policies have not adequately recognized the negative impact of land use intensification on ESV [71]. With a greater emphasis on the sustainable development of humans and the environment in the future, the protection of ecosystems will inevitably become the core of social and economic development. Therefore, we should adhere to the developmental idea of “ecological priority” and attach importance to the rational use of land to enhance ESV. This study proposes the following practical policy recommendations for the Hanjiang River Basin in Hubei Province. First, since the study area is a river basin, its regulation and support services are particularly prominent. Therefore, the sustainability of the river basin should be based on the protection of forest land and water areas [72,73]. Decision makers should increase the vegetation coverage of river basins through forest restoration and reforestation programs, increase the supervision of the aquatic environment, and moderately restore farmland to forests and grasslands. Second, due to the imbalanced regional development, counties with varying levels of socio-economic development should adopt locally differentiated regulation policies and regulation measures. For mountain counties, ecological compensation policies should be implemented to improve local economic and social conditions, while for plain counties, it is necessary to strictly control the expansion of built-up land and strengthen the protection of ecological land. It is possible to establish a long-term cross-regional ecological compensation and monitoring mechanism between mountain and plain counties. Third, to achieve the coordinated development of the socio-economy and the environment, future decision-making should incorporate ES into spatial-planning and socio-economic development policies. The ESV should be evaluated before projects progress to construction to mitigate the negative effects of human activities on ecosystems.

### 4.4. Limitations and Future Work

This study has several limitations. First, due to the opening of the middle route of the South-to-North Water Diversion Project, the natural and socio-economic environment of the HRB has been greatly affected by the change in water resources. However, the impacts of the project on local ecosystems could not be fully revealed in this study. The ESV in this study was estimated based on land use/cover data and their equivalent values proposed by Xie et al. (2003 and 2008). The change in land use/cover area cannot fully reflect the impact of the South-to-North Water Diversion Project on the ecosystem. Second, this study mainly evaluated the ecosystem as a whole, without considering the in-depth analysis of the primary and secondary services of the ecosystem. Furthermore, driving factors were selected at the macro level, such as the annual mean temperature, slope, and GDP, without considering the interactions with micro factors such as soil, the sediment concentration, microelements, etc. Our future research will improve the assessment method of ESV and evaluate ESV at the township level or grid scale [74], and the land types will be subdivided to obtain a more accurate estimation of ESV.

## 5. Conclusions

The change in ESV is the result of the joint action of natural and human forces. Exploring the temporal and spatial variation of ESV and revealing its driving factors is crucial for promoting the harmonious coexistence between human and nature. Our study analyzed how ESV changed over time due to the change in land use intensity. From 2000 to 2020, the area of built-up land increased from 2336.39 km^2^ to 3371.28 km^2^, while the area of cultivated land, grassland, and forest land decreased. The ESV of the Han River Basin in Hubei Province experienced an inverted U-shaped trend, with an increase followed by a decrease, and had the spatial distribution characteristics of high in the northwest and low in the southeast. The counties with larger forest land and water areas tended to have higher ESVs. Additionally, there was a significant negative correlation between land use intensity and ESV, which was most prominent in the northwest (LH type) and southeast (HL type) of the study area. From the analysis of the driving forces, it was found that the interaction between driving factors had a greater impact on the spatial variability of ESV than that of single factors. The spatial regression results indicated that natural factors, such as the percentage of forest land, temperature, and slope, have positive impacts on ESV, and their influence gradually increased from northwest to southeast. There was a significant spatial differentiation between socio-economic factors, i.e., both positive and negative relationships existed, and the spatial distributions of the influence coefficients were opposite to those of natural factors. In general, the influence of natural factors on ESV was greater and more significant than that of socio-economic factors, while the impact and spatial heterogeneity of socio-economic factors on ESV tended to increase. The findings in this study could provide implications for spatial planning towards promoting the sustainable development of ecosystems.

## Figures and Tables

**Table 1:** Graded assignments of land use intensity.

Types and Grades	Unused Land	Forest, Grassland, and Water Land	Agricultural Land	Urban Settlement Land
Land use types	Unused land	Forest land, Grassland, Water area	Cultivated land, Garden Land, Artificial grassland	Towns, residential areas, industrial and mining, transportation land
Grade factor	1	2	3	4

**Table 2:** Equivalent value per-unit area of ES by land use type in the HRB of Hubei Province (Unit: CNY/hm^2^).

Categories of ES	Subtypes	Cultivated Land	Forest Land	Grassland	Water Area	Unused Land	Built-Up Land
Supply services	Food production	1881.45	620.88	809.02	997.17	37.63	0.00
	Raw material production	733.77	5606.72	677.32	658.51	75.26	0.00
Regulation services	Gas regulation	1354.64	8127.87	2822.18	959.54	112.89	0.00
	Climate regulation	1825.01	7657.51	2935.06	3875.79	244.59	0.00
	Hydrological regulation	1448.72	7695.14	2859.81	35314.84	131.70	0.00
	Waste disposal	2615.22	3236.10	2483.52	27,939.55	489.18	0.00
Support services	Soil conservation	2765.73	7563.43	4214.45	771.39	319.85	0.00
	Biodiversity	1919.08	8485.34	3518.31	6453.38	752.58	0.00
Cultural services	Aesthetic landscape	319.85	3913.42	1636.86	8353.64	451.55	0.00

**Table 3:** Details of the driving factors.

Factors Type	Indicator	Description	Calculation	Reference
Natural	Temperature (X1)	Annual mean temperature (°C)	ArcGIS raster statistics	[51]
	Precipitation (X2)	Annual mean precipitation (mm)	ArcGIS raster statistics	
	Slope (X3)	Slope (°)	ArcGIS raster statistics	
	Percentage of forest land (X4)	The percentage of forest land (%)	Forest land area/total land area	
	Distance to water system (X5)	Distance to the water system (m)	ArcGIS raster statistics and Euclidean Distance	[52]
Socio-economic	GDP (X6)	GDP per unit area (10^4^ CNY/km^2^)	ArcGIS raster statistics	[51]
	Population density (X7)	Number of people per square kilometer (person/km^2^)	ArcGIS raster statistics	
	Distance to the county center (X8)	Distance to the county center (m)	ArcGIS raster statistics and Euclidean Distance	[52]
	Distance to road (X9)	Distance to road (m)	ArcGIS raster statistics and Euclidean Distance	
	Percentage of built-up land (X10)	The percentage of built-up land (%)	Built-up land area/total land area	[35]

**Table 4:** ESV of different land use types in the HRB of Hubei Province from 2000 to 2020.

Land Use Types		Cultivated Land	Forest Land	Grassland	Water Area	Unused Land	Built-Up Land	Total
2000	Areas (km^2^)	32,289.44	39,609.20	2364.14	3843.85	87.14	2336.39	80,530.16
	ESV (10^8^ CNY)	479.93	2095.58	51.91	327.97	0.23	0.00	2955.62
2010	Areas (km^2^)	31,786.21	39,546.33	2357.83	4132.90	86.27	2620.62	80,530.16
	ESV (10^8^ CNY)	472.45	2092.25	51.77	352.63	0.23	0.00	2969.34
2020	Areas (km^2^)	31,170.40	39,363.37	2334.04	4207.03	84.04	3371.28	80,530.16
	ESV (10^8^ CNY)	463.30	2082.57	51.25	358.96	0.22	0.00	2956.30
2000–2010	Areas (km^2^)	−503.23	−62.87	−6.31	289.05	−0.87	284.23	0.00
	ESV (10^8^ CNY)	−7.48	−3.33	−0.14	24.66	0.00	0.00	13.72
2010–2020	Areas (km^2^)	−615.81	−182.96	−23.79	74.13	−2.23	750.66	0.00
	ESV (10^8^ CNY)	−9.15	−9.68	−0.52	6.33	−0.01	0.00	−13.04
2000–2020	Areas (km^2^)	−1119.04	−245.83	−30.10	363.18	−3.10	1034.89	0.00
	ESV (10^8^ CNY)	−16.63	−13.01	−0.66	30.99	−0.01	0.00	0.68

**Table 5:** Factor detection results of driving factors of ESV.

	X1	X2	X3	X4	X5	X6	X7	X8	X9	X10
q statistic	0.75	0.19	0.81	0.87	0.27	0.65	0.55	0.41	0.50	0.74
*p* value	0.00 ***	0.37	0.00 ***	0.00 ***	0.13	0.00 ***	0.00 ***	0.03 **	0.00 ***	0.00 ***
rank	3	10	2	1	9	5	6	8	7	4

Note: *** and ** represent that *p* is significant at the 0.01 and 0.05 levels, respectively.

**Table 6:** Interaction detection results of driving factors of ESV.

	X1	X2	X3	X4	X5	X6	X7	X8	X9	X10
X1	0.75									
X2	0.84	0.19								
X3	0.83	0.84	0.81							
X4	0.83	0.60	0.89	0.87						
X5	0.87	0.75 ^#^	0.87	0.92	0.27					
X6	0.86	0.80	0.89	0.92	0.81	0.65				
X7	0.88	0.69	0.89	0.90	0.61	0.82	0.55			
X8	0.83	0.64	0.85	0.90	0.65	0.83	0.66	0.41		
X9	0.78	0.69	0.85	0.94	0.80	0.82	0.76	0.71	0.50	
X10	0.86	0.79	0.90	0.90	0.85	0.85	0.75	0.83	0.80	0.74

Note: ^#^ denotes nonlinear enhancement of any two factor; without ^#^ denotes enhancement of any two factor.

**Table 7:** Statistic coefficients for GWR and OLS.

	R^2^	Adjusted R^2^	AICc
GWR	0.93	0.90	49.52
OLS	0.88	0.84	248.48

## Data Availability

The data that support the findings of this study are available from the corresponding author upon reasonable request.

## Appendix A

The detailed description of the methods used in this study can be found in Appendix A.

### Appendix A.1. Methods

#### Appendix A.1.1. Hot Spot Analysis

Getis–Ord *Gi** is an index of local spatial autocorrelation used to explore the spatial clustering of high values (hot spots) or low values (cold spots) of spatial variables [48]. The output can be represented with the Z-score, *p*-value, and confidence level. We used the Getis–Ord *Gi** tool in the ArcGIS 10.3 software to analyze the hot spots and cold spots of ESV in the study area. The expression is as follows [75]:

(A1) $$G^{*}=\frac{\sum_{j=1}^{n}W_{ij}X_{j}-\bar{X}\sum_{i=1}^{n}W_{ij}}{\sqrt[{s}]{\left[n\sum_{j=1}^{n}W_{ij}{}^{2}-{\left(\sum_{j=1}^{n}W_{ij}\right)}^{2}\right]/\left(n-1\right)}}$$

(A2) $$\bar{X}=\frac{1}{n}\sum_{i=1}^{n}X_{i}$$

(A3) $$s=\sqrt{\frac{1}{n}}\sum_{i=1}^{n}X_{i}{}^{2}-\frac{1}{X^{2}}$$

where *G**** is the Z-score; *n* is the number of units; $X_{i}$ and $X_{j}$ represent the observations of variable *X* in i and j space units, respectively; $W_{ij}$ is a spatial weight matrix; and $\bar{X}$ and *s* are the average value and standard deviation, respectively. The higher the Z-score, the denser the high-values (hot spots) are, which means the higher the attribute value around the unit, and vice versa.

#### Appendix A.1.2. Bivariate Spatial Autocorrelation Model

Spatial autocorrelation refers to the statistical correlation of a certain attribute value of a geographic object with spatial location differences. Generally, the closer the two values are, the greater the correlation. Spatial autocorrelation analysis is an important indicator to measure the aggregation or discrete distribution of spatial elements, and is generally described by global Moran’s *I* and local Moran’s *I* [49]. The Moran’s *I* value is expressed as follows:

(A4) $$\mathrm{Moran}’s\;I=\frac{n\sum_{i=1}^{n}\sum_{j\neq 1}^{n}W_{ij}\cdot \left(X_{i}-\bar{X}\right)\cdot \left(X_{j}-\bar{X}\right)}{\left(\sum_{i=1}^{n}\sum_{j=1}^{n}W_{ij}\right)\sum_{i=1}^{n}{\left(X_{i}-\bar{X}\right)}^{2}}$$

where *n* is the number of the geographic unit (i.e., 39 counties in this study); $X_{i}$ and $X_{j}$ denote the actual attribute values in the sampling plots *i* and *j*, respectively; $\bar{X}$ is the average value of *X*; and $W_{ij}$ is a spatial weight matrix. When Moran’s *I* < 0, it indicates a negative correlation; when Moran’s *I* = 0, it indicates no correlation; and when Moran’s *I* > 0, it indicates a positive correlation. The greater the value, the larger the correlation between the observed values in the spatial distribution and the stronger the aggregation.

The global autocorrelation tests the spatial vergence pattern of the spatial variables over the entire research range, while the local spatial autocorrelation captures the correlation of the variables in different regional units [50]. The formula is as follows:

(A5) $$I_{kl}^{i}=z_{k}^{i}\overset{n}{\underset{j=1}{\sum }}W_{ij}z_{l}^{j}$$

where $z_{k}^{i}=\frac{X_{k}^{i}-\bar{X_{k}}}{e_{k}}$, $z_{l}^{j}=\frac{X_{l}^{i}-\bar{{\;X}_{l}}}{e_{l}}$; $X_{k}^{i}$ is the value of attribute k of sampling plot i; $X_{l}^{j}$ is the value of attribute l of sampling plot j; $\bar{X_{k}}$ and $\bar{X_{l}}$ is the average values of attributes k and l, respectively; and $e_{k}$ and $e_{l}$ are the variances of attributes k and l, respectively.

#### Appendix A.1.3. Analysis of the Driving Mechanism

##### Geographical Detector Model

The GDM comprises risk detection, factor detection, ecological detection, and interactive detection, which can be used to detect spatial variation and identify potential influencing factors [53]. The GDM has been widely used in many fields, including social economy and ecological environments [51,54]. The expression of the GDM is as follows:

(A6) $$q=1-\frac{1}{N\sigma^{2}}\overset{L}{\underset{j=1}{\sum }}N_{j}\sigma_{j}^{2}\;$$

where *q* represents the explanatory ability of the independent variable (including natural and socio-economic factors) towards the dependent variable (ESV), and *q* ∈ [0, 1]; *N* is the total sample size in the study area; *σ*^2^ is the variance; and *j* represents partition (*j* = 1,2, …, *L*). When *q* is closer to 1, it indicates that the driving factor has a greater impact on the independent variable and that the spatial heterogeneity is stronger, and vice versa.

##### Geographically Weighted Regression

The GWR is an extension of the traditional regression analysis method that can estimate data with spatial autocorrelation and reflect the spatial heterogeneity of parameters [55]. The GWR can reveal the direction and magnitude of influence of each factor in different locations [56]. The expression is as follows:

(A7) $$y_{k}=\beta_{0}\left(u_{k},v_{k}\right)+\sum_{i=1}^{n}\beta_{i}\left(u_{k},v_{k}\right)x_{ki}+c_{k}$$

where $y_{k}$ is the weighted regression value of *k*-th sample; $\beta_{0}$ is the intercept; $\left(u_{k},v_{k}\right)$ is the geographic center coordinate of the *k*-th sample; $\beta_{0}\left(u_{k},v_{k}\right)$ is the constant term; $\beta_{i}\left(u_{k},v_{k}\right)$ is the coefficient of the *k*-th independent variable of i-th driving factor; $x_{ki}$ is the *i*-th independent variable of the *k*-th sample; and $c_{k}$ is the error term. In this study, ESV is the dependent variable, and natural and socio-economic factors are the independent variables.
